# Network Representation Learning With Community Awareness and Its Applications in Brain Networks

**DOI:** 10.3389/fphys.2022.910873

**Published:** 2022-05-27

**Authors:** Min Shi, Bo Qu, Xiang Li, Cong Li

**Affiliations:** ^1^ Adaptive Networks and Control Lab, Department of Electronic Engineering, School of Information Science and Technology, Fudan University, Shanghai, China; ^2^ Peng Cheng Laboratory, Shenzhen, China; ^3^ the Institute of Complex Networks and Intelligent Systems, Shanghai Research Institute for Intelligent Autonomous Systems, Tongji University, Shanghai, China

**Keywords:** attributed networks, representation learning, community information, brain networks, node classification, link prediction

## Abstract

Previously network representation learning methods mainly focus on exploring the microscopic structure, *i.e.*, the pairwise relationship or similarity between nodes. However, the mesoscopic structure, *i.e.*, community structure, an essential property in real networks, has not been thoroughly studied in the network representation learning. We here propose a deep attributed network representation learning with community awareness (DANRL-CA) framework. Specifically, we design a neighborhood enhancement autoencoder module to capture the 2-step relations between node pairs. To explore the multi-step relations, we construct a community-aware skip-gram module based on the encoder. We introduce two variants of DANRL-CA, namely, DANRL-CA-AM and DANRL-CA-CSM, which incorporate the community information and attribute semantics into node neighbors with different methods. We compare two variant models with the state-of-the-art methods on four datasets for node classification and link prediction. Especially, we apply our models on a brain network. The superiority indicates the scalability and effectiveness of our method on various networks. Compared with DANRL-CA-AM, DANRL-CA-CSM can more flexibly coordinate the role of node attributes and community information in the process of network representation learning, and shows superiority in the networks with sparse topological structure and node attributes.

## 1 Introduction

Many real-world systems or data can be easily represented as networks. For example, in social networks, *e.g.*, Facebook Zhang et al. (2018), a node represents the user and an edge represents the friendship between users; in brain networks, *e.g.*, Fly-drosophila-medulla-1 Rossi and Ahmed (2015), a node denotes the neuron and an edge denotes the fiber tract between neurons. Obviously, networks can efficiently store and access relational knowledge between the interacting nodes Zhang et al. (2018). Hence, network analysis has been always concerned by academia and industry. Network analysis heavily relies on the network representation Wang et al. (2017). However, the traditional network representation could be sparse because it is usually developed on the discrete adjacency matrix, such as LLE Roweis and Saul (2000), Belkin and Niyogi (2001) and Ahmed et al. (2013), which would limit the expansion of the above methods in machine learning applications Perozzi et al. (2014). In recent years, network representation learning (NRL) Perozzi et al. (2014) has received widespread attention because it can learn dense and low-dimensional node embeddings with preserving the network properties. An emerging approach to NRL is based on natural language models Sheikh et al. (2019), especially skip-gram Mikolov et al. (2013a) Mikolov et al. (2013b). At present, many excellent NRL methods, such as DeepWalk Perozzi et al. (2014), node2vec Grover and Jure (2016), LINE Tang et al. (2015), GreRep Cao et al. (2015), NetMF Qiu et al. (2018) and ANE Liu et al. (2020), have been proposed.

Essentially, the above methods mainly focus on the microscopic structure that includes local closeness proximity, *i.e.*, the first-order, second-order, and high-order proximity Zhang et al. (2020), which indicate the one-step, two-step, and multi-step relations between two nodes, respectively. Notably, the community structure which is one of the common connectivity patterns observed in many kinds of networks Grover and Jure (2016), can reveal the implicit relationships between nodes from a higher structural level over the microscopic structure Wang et al. (2017) Keikha et al. (2018). For example, communities in functional brain networks are likely to group brain regions having similar functions Sporns and Betzel (2016) Wang and Li (2019). The algorithms Wang et al. (2017) Keikha et al. (2018) have proved that incorporating the community information into NRL is conducive to learn discriminative node representations. To further capturing the highly non-linearity that is universal in networks Wang et al. (2016) Gao and Huang (2018) Zheng et al. (2020), deep learning technologies have been applied in NRL, such as community-based variational autoencoder (ComVAE) Shi et al. (2019).

Moreover, nodes are often accompanied with a rich set of attributes. Sheikh *et al.* Sheikh et al. (2019) state that the attributes are invaluable when structural information is missing or structurally unrelated nodes have high attribute similarity. Currently, some attributed NRL methods Zhang et al. (2018) Gao and Huang (2018) Zheng et al. (2020) Liao et al. (2018) Yang et al. (2015) Huang et al. (2017a) have been designed to integrate the structure and the attributes in a principled way.

Evidently, mining the community information and preserving the attribute semantics are both advantageous to enhance the quality of node embeddings based on the microscopic structure. Therefore, Zhu *et al.* Zhu et al. (2018) propose the CTDW algorithm.

However, how to effectively integrate the multiple heterogeneous information sources, including the microscopic structure, community structure and attribute semantics, from non-linear relational data for learning informative network representation is still challenging.

Therefore, we propose a deep coupling framework in the paper, *i.e.*, deep attributed network representation learning with community awareness (DANRL-CA). Precisely, the DANRL-CA framework consists of two coupled modules, that is, the neighborhood enhancement autoencoder and community-aware skip-gram, which share connections to the encoder. In particular, the community information and attribute information are preprocessed, and then integrated into the adjacency matrix as the input of the framework.

To summarize, we make the following contributions:

• We propose a deep coupling DANRL-CA framework. To preserve the second-order proximity, the neighborhood enhancement autoencoder module reconstructs the target neighbors of nodes. The target neighbors are obtained by incorporating the community information and attribute information into the adjacency matrix. To capture the high-order proximity, we design a community-aware skip-gram module based on the encoder.
• We preprocess the community information in two ways. One is to treat the community information as node attributes. The other is to calculate the community similarity matrix on the assumption that the representations of nodes within a community should be more similar than those belonging to different communities. Then, we define two variants of DANRL-CA, namely, the DANRL-CA-AM and DANRL-CA-CSM.
• Compared with DANRL-CA-AM, the DANRL-CA-CSM model mostly shows superior performance on four datasets and two network analysis tasks, which is explained that DANRL-CA-CSM can flexibly balance the contribution of attribute semantics and community information to the quality of network representation. Moreover, DANRL-CA-CSM has a better representation for the networks with sparse network structure and node attributes over DANRL-CA-AM.
• The proposed method DANRL-CA has excellent performance on brain networks without node attribute information, which shows that our methods can still be extended to networks with only structural information, and have prospects for application in brain science.

The rest of the paper is organized as follows. In Section 2, we review the related work. The preliminaries involved in the paper are given in Section 3. In Section 4, we detail the proposed DANRL-CA framework. In Section 5 and 6, we introduce how we conduct the experiments, and discuss the experimental results. Finally, we conclude our work in Section 7.

## 2 Related Work

In recent years, NRL as an effective feature mining method has achieved extensive attention. The success of natural language models provides a new direction for NRL Perozzi et al. (2014), Grover and Jure (2016), Tang et al. (2015), Cao et al. (2015), Qiu et al. (2018), Liu et al. (2020). The above methods are on the assumption that the nodes with similar contexts (sequences) in the structure also have similar representations in the new vector space. However, social information networks in the real world are usually sparse, which could result in poor node embeddings Zhang et al. (2016).

Significantly, a node is usually accompanied by auxiliary information, which can be defined as node attributes. The attributes can reflect and affect the community structure of networks Marsden and Friedkin (1993) McPherson and Cook (2001) Marsden (1988). Based on the strong correlation of the structure and the attributes, the representation of attribute networks has been vigorously explored Gao and Huang (2018) Zheng et al. (2020) Liao et al. (2018) Yang et al. (2015) Huang et al. (2017a). Moreover, how to jointly embed the heterogeneous information sources is a challenging task. The previous work Zhang et al. (2018) shows that the deep coupling paradigm is beneficial to integrate the multiple information sources from complex networks to learn robust node representations. Specifically, ANRL is a deep two-part coupling model, which is composed of neighbor enhancement autoencoder and attribute-aware skip-gram module. The two modules share connections to the encoder.

However, most of the previous attributed NRL methods merely consider the microscopic structure. Wang *et al.* Wang et al. (2017) point out that for two nodes within a community, even if they only have a weak relationship in the microscopic structure due to the data sparsity issue, their similarity will also be strengthened by the community structure constraint. Hence, Zhu *et al.* Zhu et al. (2018) propose CTDW, which incorporates the community features and text features of nodes into NRL under the framework of matrix factorization. Nevertheless, the design of matrix factorization requires a high computational cost. Meanwhile, the creation of shallow model Hamilton et al. (2017) restricts the representation ability of CTDW for complex networks.

To integrate the multiple heterogeneous information sources, *i.e.*, the community information, attribute semantics, and microscopic structural information, from the non-linear attributed network data, and then learn scalable and effective network representation, we propose a deep coupling neural network framework, *i.e.*, DANRL-CA, in which the neighborhood enhancement autoencoder and community-aware skip-gram module are tightly interconnected as they share the first several layers. Notably, the community information and attribute information are incorporated into the adjacency matrix to enhance the direct neighborhood of nodes.

## 3 Preliminaries

In this section, we first give some notations and network properties involved in the paper, and then declare the formal definition of the problem to be solved.

### 3.1 Notations

Let *G* = (*V*, *E*, *A*, *X*) be an attributed social information network, where *V* = {*v*_1_, … , *v*_*n*_} is the set of *n* nodes, *E* ⊂ (*V* × *V*) is the set of edges, *A* denotes the adjacency matrix and *X* represents the attribute matrix. In the adjacency matrix *A*, if the network is undirected, *a*_*ij*_ = *a*_*ji*_. If the network is unweighted, an edge exists between nodes *v*_*i*_ and *v*_*j*_, *a*_*ij*_ = 1, or else *a*_*ij*_ = 0. The row *X*_*i*_ in the attribute matrix *X* denotes the attribute information associated with node *v*_*i*_. Here, we discuss the undirected and unweighted networks.

### 3.2 Network Properties

1) Community information

The community matrix $C\in R^{n\times l}$ , where *l* indicates the number of communities, can be obtained through some non-overlapping community detection methods. If node *v*_*i*_ belongs to the community *m*, the corresponding element *c*_*im*_ = 1, else *c*_*im*_ = 0.

2) Attribute proximity

The attribute proximity denotes the proximity between node pairs that are evidenced by the attributes. Specifically, the attribute proximity between nodes *v*_*i*_ and *v*_*j*_ is determined by the similarity between *X*_*i*_ and *X*_*j*_.

3) Second-order proximity and High-order proximity.

The second-order proximity and high-order proximity both indicate the indirect proximity between nodes *v*_*i*_ and *v*_*j*_, which is because node *v*_*j*_ is within the context of node *v*_*i*_ instead of an edge between them. The second-order proximity captures the 2-step relations between each pair of nodes, which can be determined by the number of common neighbors shared by node pairs Zhang et al. (2020). The high-order proximity explores the *k*-step (*k* ≥ 3) relations, which can be reflected by the number of *k*-step (*k* ≥ 3) paths from node *v*_*i*_ to node *v*_*j*_ Zhang et al. (2020).

### 3.3 Attributed Network Representation Learning

Given an attributed social information network *G* = (*V*, *E*, *A*, *X*), we aim at embedding the network into a new low-dimensional vector space via learning a mapping function $f:G\to Y\in R^{n\times d}$ , where *d* (≪ *n*) is the dimension of network representation. Then, each node can be represented with a vector. The objective of the function is to preserve the structure and attribute information simultaneously.

## 4 Methods

In this section, an overview of the proposed DANRL-CA framework is provided. Then, we describe the selected community detection algorithms and how we preprocess the community information and attribute information in two variant models. Next, we introduce the framework design in detail. Finally, we give the optimization of the models.

### 4.1 Overview

**FIGURE 1:**
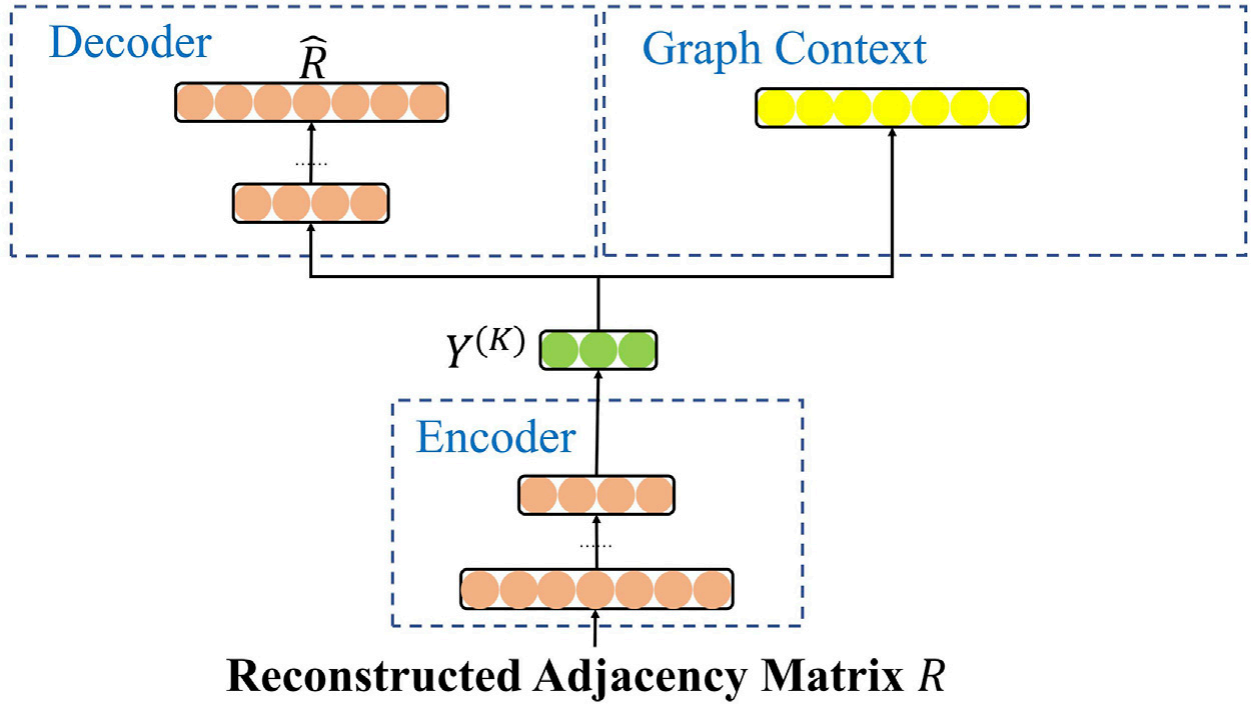
The architecture of the proposed DANRL-CA framework.

The DANRL-CA framework takes the encoder component as the basis to extend two branches, which are used to preserve the second-order and high-order proximity, respectively. Figure 1 shows the architecture of the DANRL-CA framework. In the framework, the encoder and decoder component build the neighborhood enhancement autoencoder module, and the encoder and graph context component construct the community-aware skip-gram module. Motivated by ComVAE Shi et al. (2019), we first modularize the community detection algorithms to mine the optimal community information on the networks. Then, the community information, attribute semantics, and adjacency matrix are aggregated (details see Section 4.3), which is the input, namely, the reconstructed adjacency matrix *R*, of the DANRL-CA. In particular, based on different algorithms to process community information, we design two variants of the framework, namely, the DANRL-CA-AM model and the DANRL-CA-CSM model.

### 4.2 Community Detection Algorithms

Taking the applicability of classical community detection algorithms in the large-scale networks into account Zhao et al. (2017), three effective methods, *i.e.*, Label Propagation Algorithm (LPA) Raghavan et al. (2007), Infomap Rosvall and Bergstrom (2008), and Multilevel Blondel et al. (2008), are employed.

1) Label Propagation Algorithm (LPA)

By allocating each node with a unique community label as the initialization, LPA merges the community label of each node through the voting of node neighbors until convergence Shi et al. (2019). The computational complexity of LPA is a linear function of the number of edges, *i.e.*, O(E) .

2) Infomap

Infomap encodes node sequences with the shortest length based on the information theory, and then detects the communities through a deterministic greed search strategy Shi et al. (2019). Primarily, the node sequences are obtained by random walk sampling. Infomap runs in O(E) .

3) Multilevel

Multilevel is divided into two phases that are repeated iteratively until the modularity cannot be increased. The first phase is that after assigning a distinct community for each node, a node is moved to the community of one of its neighbors based on the highest positive contribution to modularity, which is repeated for all nodes until there is no further improvement. The second phase is that each community itself is considered as a node. The computational complexity of Multilevel is O(NlogN) .

### 4.3 Preprocessing

In this part, we describe the preprocessing of community information and attribute information in detail.

1) Community information

We deal with the community information in two ways.

• **DANRL-CA-AM:** If categorizing the community information as node attributes, we will obtain a new attribute matrix *W*, which is constructed by concatenating the attribute matrix *X* and community matrix *C*. Here, the community matrix *C* is mined by the above community detection algorithms, where rows identify all nodes and columns list all community categories. When node *v*_*i*_ is located in the community *m*, the element in the *i*th row and the *m*th column is 1, otherwise it is 0.
• **DANRL-CA-CSM:** The community information implies that the representations between the target node and its neighbors within a community could be similar. To capture the characteristic, we calculate a community similarity matrix *C* ^(*S*)^, which is used as the similarity measurement method. Each element $c_{ij}^{\left(S\right)}$ of the community similarity matrix is defined as

$$c_{ij}^{\left(S\right)}=CosineSimilarity\left(C_{i},C_{j}\right)=\frac{C_{i}C_{j}^{T}}{\left|C_{i}\|C_{j}\right|},$$ (1)

where *C*_*i*_ denotes the community information related to node *v*_*i*_.

2) Attribute information

Similarly, we obtain an attribute similarity matrix, which is on the observation of social homophily Marsden and Friedkin (1993) McPherson and Cook (2001), and the description of each element for the two variant models is shown below respectively.

In the DANRL-CA-AM model,

$$w_{ij}^{\left(S\right)}=CosineSimilarity\left(W_{i},W_{j}\right)=\frac{W_{i}W_{j}^{T}}{\left|W_{i}\|W_{j}\right|}.$$ (2)

Here, *W*_*i*_ indicates the reorganized attribute information related to node *v*_*i*_.

In the DANRL-CA-CSM model,

$$x_{ij}^{\left(S\right)}=CosineSimilarity\left(X_{i},X_{j}\right)=\frac{X_{i}X_{j}^{T}}{\left|X_{i}\|X_{j}\right|}.$$ (3)

Here, *X*_*i*_ represents the original attribute information affiliated with node *v*_*i*_.

Most of the networks in the real world are sparse Zhang et al. (2016). The more the common neighbors between node pairs are, the more accurate the preserved second-order proximity is. Therefore, we propose to linearly combine the similarity matrix and adjacency matrix via setting the hyperparameters. The result is seen as the reconstructed adjacency matrix *R*, which is given below for the DANRL-CA models.

In the DANRL-CA-AM model,

$$R=\eta A+\psi W^{\left(S\right)}.$$ (4)

Here, *A* is the adjacency matrix, *W* ^(*S*)^ denotes the reorganized attribute similarity matrix, and the hyperparameters *η* and *ψ* are used to control the effect of *A* and *W* ^(*S*)^ separately.

In the DANRL-CA-CSM model,

$$R=\eta A+\psi X^{\left(S\right)}+\phi C^{\left(S\right)}.$$ (5)

Here, *A* represents the adjacency matrix, *X* ^(*S*)^ is the original attribute similarity matrix, and *C* ^(*S*)^ is the community similarity matrix. Similarly, the hyperparameters *η*, *ψ* and *ϕ* aim at balancing the roles of the above matrices, respectively.

### 4.4 Framework Design

In this section, the architecture of the proposed DANRL-CA framework is introduced, including the neighborhood enhancement autoencoder module and the community-aware skip-gram module.

1) Neighborhood enhancement autoencoder module.

The reconstruction criterion of the autoencoder is to capture the data manifolds smoothly and thus preserve the similarity between samples Salakhutdinov and Hinton (2009). This feature of the autoencoder is beneficial for preserving the second-order proximity. When the reconstructed adjacency matrix *R* is taken as the input of the autoencoder, each instance *R*_*i*_ characterizes the neighborhood structure with the community information and attribute semantics of the corresponding node *v*_*i*_. Then, the reconstruction process of the autoencoder could make the nodes with similar neighborhood structure also have similar latent representations. Hence, the neighborhood enhancement autoencoder module is proposed in the DANRL-CA framework.

The autoencoder consists of the encoder and decoder. Next, we give the relationship between the input and output of each layer in the encoder

$$\begin{array}{ll} y_{i}^{\left(1\right)} & =\delta \left(R_{i}W^{\left(1\right)}+b^{\left(1\right)}\right)\\ y_{i}^{\left(k\right)} & =\delta \left(y_{i}^{\left(k-1\right)}W^{\left(k\right)}+b^{\left(k\right)}\right),k\in \left\{2,\ldots ,K\right\}, \end{array}$$ (6)

where *R*_*i*_ is the *i*th row data in the reconstructed adjacency matrix *R*. The symbol *δ*(.) denotes the non-linear activation functions, which is typically the elementwise sigmoid or hyperbolic tangent nonlinearity (tanh), or the identity function if staying linear Bengio et al. (2013). Furthermore, the parameters *W* ^(*k*)^ and *b* ^(*k*)^ indicate the weight matrix and bias vector in the *k*th layer, respectively, and *K* represents the number of layers.

The decoder is the inverse calculation process of the encoder, which here shares the same activation function with the encoder and is designed to obtain the reconstructed output $\hat{R}$ of input *R*.

Then, by minimizing the error between the input and output, the loss function of the autoencoder is defined as

$$L_{ae}=\overset{n}{\underset{i=1}{\sum }}L_{i}=\overset{n}{\underset{i=1}{\sum }}\|\hat{R_{i}}-R_{i}{\|}_{2}^{2},$$ (7)

where *n* is the number of nodes.

Significantly, as shown in Bengio et al. (2013), the choice of activation function *δ*(.) in the decoder depends largely on the input domain range and nature and is usually chosen so that *L*_*i*_ returns a negative log likelihood for the observed value of *R*_*i*_. Hence, in the paper, we choose the tanh function.

Inspired by SDNE Wang et al. (2016), to capture the meaningful edge information effectively, we impose more penalty to the reconstruction error of non-zero elements than that of zero elements, and the modified objective function is shown as

$$L_{ae}^{M}=\overset{n}{\underset{i=1}{\sum }}\|\left(\hat{R_{i}}-R_{i}\right)⊙b_{i}{\|}_{2}^{2},$$ (8)

where ⊙ means the Hadamard product and $b_{i}={\left\{b_{ij}\right\}}_{j=1}^{n}$ . If the element *r*_*ij*_ = 0, *b*_*ij*_ = 1, else *b*_*ij*_ = *χ* > 1.

2) Community-aware skip-gram module

We use the encoder, which encodes the community information and node attributes into network representation, to replace the input and hidden layer of the classic three-layer neural network skip-gram, and then design the community-aware skip-gram module. Inspired by the excellent performance of Deepwalk on sparse networks, we also train the skip-gram module by node sequences. We adopt the alias node sampling strategy and objective optimization with negative sampling to speed the training, which are introduced in node2vec. The corresponding objective of the community-aware skip-gram module is expressed as Equation 9

$$\begin{array}{ll} L_{sg}^{NS} & =-\overset{n}{\underset{i=1}{\sum }}\underset{c\in C}{\sum }\underset{-b\leq j\leq b,j\neq 0}{\sum }\{\log\sigma \left({h\prime }_{\left(i+j\right)}^{T}y_{i}^{{\left(K\right)}^{T}}\right)\\ & \left.\;+\overset{\left|neg\right|}{\underset{s=1}{\sum }}E_{v_{s}∼P_{n}\left(v\right)}\left[\log\sigma \left(-h_{s}^{\prime T}y_{i}^{{\left(K\right)}^{T}}\right)\right]\right\}, \end{array}$$ (9)

where *n* is the number of nodes in the networks, *c* ∈ *C* denotes the sampled node sequences, and *b* is the window size. The symbol $h_{i}^{\prime }$ is the *i*th column data of the transition matrix *H*′ between the middle representation layer of the autoencoder and output layer of the skip-gram, $y_{i}^{\left(K\right)}$ denotes the representation of node *v*_*i*_, $\sigma \left(x\right)=\frac{1}{1+\exp\left(-x\right)}$ is a sigmoid activation function, and |*neg*| indicates the number of negative samples. The sampling distribution $P_{n}\left(v\right)\propto d_{v}^{3/4}$ is set as suggested in Mikolov et al. (2013b), where *d*_*v*_ represents the degree of node *v*_*n*_, and $E_{v_{s}∼P_{n}\left(v\right)}$ indicates that the noise node *v*_*s*_ is expected to be sampled based on the probability distribution *P*_*n*_(*v*).

The community-aware skip-gram module aims at capturing the high-order proximity.

### 4.5 Model Optimization

To learn scalable and effective node representations, we combine the loss of the community-aware skip-gram module and the neighborhood enhancement autoencoder module by the hyperparameter *α* that is used to balance the contribution of two branches

$$L_{c}=L_{sg}^{NS}+\alpha L_{ae}^{M}.$$ (10)

However, overfitting may occur due to the employment of the autoencoder. To alleviate the phenomenon, we add the *l*_2_ norm regularizer

$$L_{\mathrm{reg}}=\frac{1}{2}\overset{K}{\underset{k=1}{\sum }}\left(\|W^{\left(k\right)}{\|}_{F}^{2}+\|{\hat{W}}^{\left(k\right)}{\|}_{F}^{2}\right),$$ (11)

where *K* is the number of layers in the encoder and decoder. The symbols *W* ^(*k*)^ and ${\hat{W}}^{\left(k\right)}$ are used to represent the weight matrix of the encoder and decoder in the *k*th layer, respectively.

Overall, we optimize the following loss function

$$L=L_{sg}^{NS}+\alpha L_{ae}^{M}+\gamma L_{\mathrm{reg}},$$ (12)

where *γ* is the coefficient of *L*_*reg*_.

For each variant model, by iteratively training two modules until the entire model converges, we learn informative node embeddings, *i.e.*, the representation output *Y* ^(*K*)^ of the autoencoder.

Algorithm 1 describes the learning process of the entire framework, and all parameters are denoted as *Θ*.

Algorithm 1 Framework of DANRL-CA

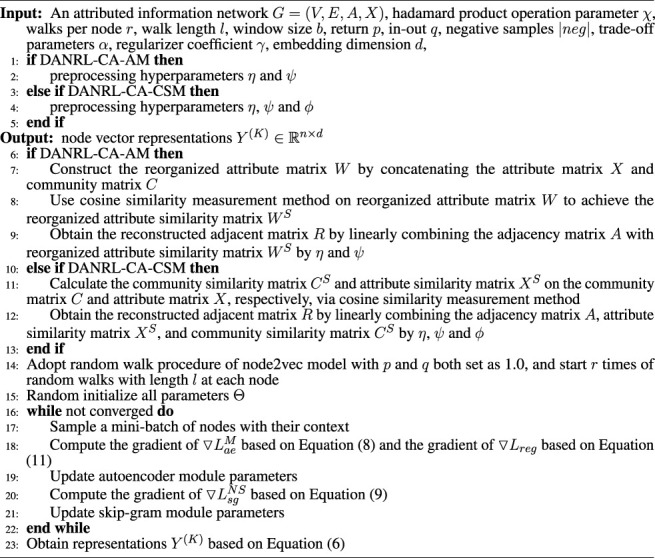

## 5 Materials

In this section, we provide an overview of the datasets and baselines used in our experiments. In addition, we introduce the experimental setup. The validity of the proposed models over other state-of-the-art methods is assessed via two machine learning tasks, namely, node classification Sen et al. (2008) Kazienko and Kajdanowicz (2012) and link prediction Wang et al. (2020) Mallick et al. (2019). Specially, the proposed models are also applied on brain networks, and perform well in link prediction.

### 5.1 Datasets

The experiments operate on Citeseer Zhang et al. (2018), PubMed Zhang et al. (2018), Cora Liu et al. (2019), Flickr Huang et al. (2017b), and Fly-drosophila-medulla-1 Rossi and Ahmed (2015) datasets, where the first three belong to citation networks, and the last two belong to the social network and the brain network respectively. Table 1 summarizes the statistics of datasets. Meanwhile, we give an introduction to the above datasets as follows.

1) Citeseer: In this dataset, the papers are classified into Agents, AI, DB, IR, ML and HCI, where the six classes are as node labels. In addition, a node and an edge indicate the paper and the citation relation between papers, separately. After removing the stop-words and the words that appear less than 10 times in the paper, the remaining unique words are as node attributes.
2) PubMed: This is a citation network. The papers are divided into three classes: Diabetes Mellitus Experimental, Diabetes Mellitus Type 1, and Diabetes Mellitus Type 2, which are regarded as the labels of nodes. The papers are described by TF/IDF weighted word vectors in a dictionary composed of unique words, *i.e.*, node attributes, on diabetes from the PubMed database.
3) Cora: This citation dataset consists of machine learning papers that are classified into seven classes, namely, Case Based, Genetic Algorithms, Neural Networks, Probabilistic Methods, Reinforcement Learning, Rule Learning and Theory. The classes are again viewed as node labels. Moreover, the bag-of-words model is used to deal with the papers, and node attributes are obtained.
4) Flickr: Flickr is an image hosting and sharing website. In this dataset, a node represents the user, and an edge represents the friendship between users. Moreover, a list of tags used to describe the interests of users is treated as node attributes, and the pre-defined photo groups are regarded as node labels.
5) Fly-drosophila-medulla-1: The dataset reveals the nerve fiber network of fly *drosophila* medulla, where a node denotes the neuron and an edge describes the fiber tract between neurons.

**TABLE 1:** Dataset statistics.

Datasets	*#* Nodes	*#* Edges	*#* Attributes	*#* Labels
Citeseer	3,312	4,714	3,703	6
PubMed	19,717	44,338	500	3
Cora	2,708	5,429	1,433	7
Flickr	7,575	239,738	12,047	9
Fly-drosophila-medulla-1	1,781	9,016	—	—

### 5.2 Baselines

To evaluate the performance of the proposed DANRL-CA models, we compare them with seven algorithms, including six structure-based methods, *i.e.*, M-NMF Wang et al. (2017), DeepWalk Perozzi et al. (2014), node2vec Grover and Jure (2016), LINE Tang et al. (2015), SDNE Wang et al. (2016), and ComVAE Shi et al. (2019), in which M-NMF, and ComVAE both consider incorporating the community information into NRL, and one structure and attribute based method, namely, ANRL Zhang et al. (2018).

1) DeepWalk: DeepWalk uses the uniform random walk to sample the linear node sequences, which preserves the high-order proximity.
2) node2vec: Unlike the rigid sampling strategy of DeepWalk, node2vec utilizes the biased random walk to explore a more flexible neighborhood structure.
3) LINE: LINE defines the clear objective function for capturing the first-order and second-order proximity.
4) SDNE: SDNE is a deep model with a semi-supervised architecture, in which the supervised component exploits the first-order proximity and the unsupervised component reconstructs the second-order proximity.
5) M-NMF: NMF is a matrix factorization method that decomposes a non-negative matrix into the product of two non-negative matrices, which aims at obtaining the dimensionality reduction matrix of data features. Specifically, M-NMF applies the NMF based learning module to incorporate the first-order and second-order proximity, and the modularity-based community detection module to obtain the community information. Then, M-NMF exploits the consensus relationship from the two modules for learning network representation.
6) ComVAE: ComVAE contains two main modules, i.e., the community detection module and the deep learning module. The community detection module is to obtain the community information. The deep learning module is to integrate the second-order proximity and community information for robust node representations. Here, the community detection methods, Infomap and LPA, are applied.
7) ANRL: ANRL takes the encoder component as a basis to extend two modules, namely, the neighbor enhancement autoencoder and attribute-aware skip-gram, which intend to capture the second-order and high-order proximity from the structure, respectively. Furthermore, in the original paper, the comparison of experimental results among the variants of ANRL shows that ANRL-WAN has the optimal performance. Hence, we select ANRL-WAN as our baseline.

### 5.3 Experimental Setup

For all baselines, we adopt the implementation released by the original authors, and tune the parameters to make the models get the best performance. Especially, for M-NMF, to construct the community indicator matrix and community representation matrix, the parameter *k* is set as the actual number of communities on datasets. For LINE, we concatenate the first-order and second-order representation of each node to achieve the final node representations. In the experiments, the dimension *d* of network representation is set as 128. Furthermore, we set walks per node *r* as 10, walk length *l* as 80, window size *b* as 10, negative samples |*neg*| as 10, return *p* and in-out *q* both as 1.0 of our models. The hyperparameters *η*, *ψ*, *ϕ*, *χ*, *α* and *γ* are tuned by using the grid search, in which the preprocessing hyperparameters *η*, *ψ* and *ϕ* take $\left\{0.5,1.0,1.5,2.0,2.5\right\}$ as a benchmark, and then are fine-tuned according to the actual situation. He *et al.* point out that stacking multiple non-linear layers helps to learning better representations He et al. (2016). Particularly, the tower structure design with halved layer size for each successive higher layer, which is followed by hidden layers component, has been shown to be effective by recent work Liao et al. (2018). Inspired by the above, and according to the comparison of experimental results under different designs, Tables 2–7 show the neural network architecture of the proposed DANRL-CA models on the Citeseer, PubMed, Cora, and Flickr datasets, and two tasks, *i.e.*, node classification (NC) and link prediction (LP). Especially, for the Fly-drosophila-medulla-1 dataset, there are no node label information and node attribute information. Here, we perform experiments on link prediction, and do not discuss the model construction of DANRL-CA-AM and DANRL-CA-CSM separately. The corresponding description is illustrated in Table 8.

**TABLE 2:** Detailed architecture information for datasets (***DANRL-CA-AM/LPA***).

Datasets	*#* Neurons in Each Layer
Citeseer	3312-1000-500-128-500-1000-3312
PubMed	19717-1000-500-128-500-1000-19717
Cora	2708-1000-500-128-500-1000-2708
Flickr	7575-500-128-500-7575 (NC)
	7575-1000-500-128-500-1000-7575 (LP)

**TABLE 3:** Detailed architecture information for datasets (***DANRL-CA-AM/Infomap***).

Datasets	*#* Neurons in Each Layer
Citeseer	3312-1000-500-128-500-1000-3312
PubMed	19717-500-128-500-19717 (NC)
	19717-1000-500-128-500-1000-19717 (LP)
Cora	2708-1000-500-128-500-1000-2708
Flickr	7575-500-128-500-7575 (NC)
	7575-1000-500-128-500-1000-7575 (LP)

**TABLE 4:** Detailed architecture information for datasets (***DANRL-CA-AM/Multilevel***).

Datasets	*#* Neurons in Each Layer
Citeseer	3312-1000-500-128-500-1000-3312 (NC)
	3312-500-128-500-3312 (LP)
PubMed	19717-256-128-256-19717 (NC)
	19717-500-128-500-19717 (LP)
Cora	2708-1000-500-128-500-1000-2708 (NC)
	2708-500-128-500-2708 (LP)
Flickr	7575-500-128-500-7575 (NC)
	7575-1000-500-128-500-1000-7575 (LP)

**TABLE 5:** Detailed architecture information for datasets (***DANRL-CA-CSM/LPA***).

Datasets	*#* Neurons in Each Layer
Citeseer	3312-1000-500-128-500-1000-3312
PubMed	19717-1000-500-128-500-1000-19717
Cora	2708-2000-1000-500-128-500-1000-2000-2708
Flickr	7575-256-128-256-7575 (NC)
	7575-1000-500-128-500-1000-7575 (LP)

**TABLE 6:** Detailed architecture information for datasets (***DANRL-CA-CSM/Infomap***).

Datasets	*#* Neurons in Each Layer
Citeseer	3312-1000-500-128-500-1000-3312 (NC)
	3312-256-128-256-3312 (LP)
PubMed	19717-1000-500-128-500-1000-19717
Cora	2708-1000-500-128-500-1000-2708 (NC)
	2708-256-128-256-2708 (LP)
Flickr	7575-1000-500-128-500-1000-7575

**TABLE 7:** Detailed architecture information for datasets (***DANRL-CA-CSM/Multilevel***).

Datasets	*#* Neurons in Each Layer
Citeseer	3312-2000-1000-500-128-500-1000-2000-3312 (NC)
	3312-500-128-500-3312 (LP)
PubMed	19717-1000-500-128-500-1000-19717
Cora	2708-2000-1000-500-128-500-1000-2000-2708 (NC)
	2708-1000-500-128-500-1000-2708 (LP)
Flickr	7575-500-128-500-7575 (NC)
	7575-1000-500-128-500-1000-7575 (LP)

**TABLE 8:** Detailed architecture information for datasets (***DANRL-CA/LPA/Infomap/Multilevel***).

Datasets	*#* Neurons in Each Layer
Fly-drosophila-medulla-1 (LPA)	1781-1000-500-128-500-1000-1781 (LP)
Fly-drosophila-medulla-1 (Infomap)	1781-500-128-500-1781 (LP)
Fly-drosophila-medulla-1 (Multilevel)	1781-500-128-500-1781 (LP)

## 6 Results and Discussion

### 6.1 Citation Networks and Social Network

1) Node Classification

Node classification is usually used for labeling data, which is a significant task in reality. In the experiment, we utilize SVM as the classifier, and use Micro-F1 and Macro-F1 as the metrics of evaluating multi-label classification results. Specifically, the node representations are first learned. Then, we randomly sample 30% of the labeled nodes as the training data, and use the left to test the performance. To reduce the influence of the randomness, which is of the initial values of the classifier parameters, on the experimental results, we repeat the process 10 times, and calculate the average performance as the final results as Zhang et al. (2018) Zhang et al. (2020) Liao et al. (2018) done. Table 9 shows the performance comparison. Next, we summarize and analyze the observations.

**TABLE 9:** Node classification results on Citeseer, Pubmed, Cora, BlogCatalog and Flickr datasets.

Datasets	Citeseer	PubMed	Cora	Flickr
Evaluation	Micro-F1 Macro-F1	Micro-F1 Macro-F1	Micro-F1 Macro-F1	Micro-F1 Macro-F1
DeepWalk	0.5665 0.5212	0.8109 0.7978	0.7900 0.7782	0.4940 0.4835
node2vec	0.6002 0.5465	0.8104 0.7968	0.8058 0.7942	0.5155 0.5062
LINE	0.5605 0.5256	0.8049 0.7926	0.7884 0.7767	0.5613 0.5576
SDNE	0.4161 0.3632	0.4258 0.2900	0.5813 0.5201	0.6043 0.5991
M-NMF	0.5337 0.4814	0.7175 0.6630	0.6416 0.6269	0.6028 0.5974
ComVAE (Infomap)	0.2189 0.1521	0.3944 0.2990	0.2527 0.1372	0.5167 0.5095
ComVAE (LPA)	0.2173 0.1580	0.3952 0.2997	0.2416 0.1337	0.5383 0.5299
ANRL-WAN	**0.7246 0.6764**	0.8595 0.8584	0.8161 0.8030	0.6701 0.6584
DANRL-CA-AM/Infomap	0.7154 0.6658	0.8583 0.8551	0.8324 0.8204	**0.9135 0.9125**
DANRL-CA-AM/LPA	0.7138 0.6710	0.8452 0.8421	0.8350 **0.8228**	0.9128 0.9118
DANRL-CA-AM/Multilevel	0.7146 0.6739	0.8189 0.8125	**0.8358** 0.8225	0.9002 0.8988
DANRL-CA-CSM/Infomap	0.7155 0.6631	0.8753 0.8740	0.8313 0.8173	0.9057 0.9042
DANRL-CA-CSM/LPA	0.7122 **0.6750**	0.8774 0.8751	0.8313 0.8166	0.9056 0.9043
DANRL-CA-CSM/Multilevel	**0.7177** 0.6723	**0.8791 0.8772**	0.8336 0.8193	0.9078 0.9065

⋆ We use red bold to highlight the best performance, and utilize black bold to show the performance comparison results between DANRL-CA-AM, and DANRL-CA-CSM, respectively. Significantly, there is the overlap between the red bold part and the black bold part.

Notably, M-NMF and DANRL-CA consider the preservation of community information more than LINE and ANRL-WAN, respectively. Different from the superiority of LINE over M-NMF on most datasets, the proposed DANRL-CA models show better classification results over ANRL-WAN on almost all datasets, which shows that how to integrate the community information and microscopic structural information in principle is essential, and proves that the introduction of community information is meaningful for learning network representation indeed. We also see that the models considering both the structure and the attributes consistently outperform those only focusing on the structure, and the gap is more evident on the social network with rich attribute information. The above observation suggests that the reasonable integration of the structure and the attributes facilitates the learning of accurate network representation. Based on all the above discussions, the proposed DANRL-CA models show the best performance in almost all cases, demonstrating the scalability and effectiveness of our method.

Next, we compare DANRL-CA-AM and DANRL-CA-CSM, which shows that DANRL-CA-CSM mostly has superior performance. The observation explains that the design of DANRL-CA-CSM can flexibly adjust the positive effect of attribute semantics and community information on the accuracy of node representations. Notably, the significant difference in the PubMed dataset further reveals that when the network structure and the node attributes are sparse, the community information will greatly affect the representation of the network, which is because as shown in Table 1, PubMed has the most sparse topological structure and the fewest attribute tags in all datasets.

Furthermore, we execute the performance comparison under the different community detection methods about ComVAE, DANRL-CA-AM, and DANRL-CA-CSM. The result shows that the accuracy of community information will affect the performance of the model, which suggests that it is meaningful to modularize the community detection methods because the way is advantageous to improve the flexibility and applicability of the model on various networks.

2) Link Prediction

Link prediction is a connectivity prediction task, which aims to infer the missing and/or false edges, or predict the nonexistent edges that are likely to generate in the future Zhang et al. (2020). In our work, to obtain the ground truth, 50% of edges are removed from the original network, and the selected models embed the new network. Note that the remaining network is guaranteed to be connected while the edges are removed. The removed edges are regarded as positive samples. We randomly sample the same number of nonexistent edges from the original network, which are used as negative samples. Then, the positive and negative samples constitute the test set. We rank both the positive and negative samples under the similarity calculation about node representations based on the cosine similarity function, and utilize the AUC Fawcett (2006) index to evaluate the ranking quality. Table 10 shows the results. Obviously, the higher the score is, the better the performance of the model is. Next, we have the discussions below.

**TABLE 10:** Link prediction results on Citeseer, Pubmed, Cora, BlogCatalog and Flickr datasets.

Datasets	Citeseer	PubMed	Cora	Flickr
Evaluation	AUC	AUC	AUC	AUC
DeepWalk	0.6020	0.7925	0.7209	0.7247
node2vec	0.5485	0.7977	0.7244	0.7341
LINE	0.5309	0.6213	0.6047	0.5262
SDNE	0.6093	0.7562	0.6326	0.9023
M-NMF	0.6249	0.7944	0.7884	0.8725
ComVAE (Infomap)	0.5729	0.5531	0.5703	0.7635
ComVAE (LPA)	0.5654	0.5518	0.5727	0.7539
ANRL-WAN	**0.9666**	0.8035	0.9181	0.7800
DANRL-CA-AM/Infomap	0.9562	0.8700	0.9246	**0.9383**
DANRL-CA-AM/LPA	0.9550	0.8981	0.9314	0.9377
DANRL-CA-AM/Multilevel	0.9531	0.8414	0.9244	0.9382
DANRL-CA-CSM/Infomap	0.9565	**0.9592**	0.9276	0.9378
DANRL-CA-CSM/LPA	0.9528	0.9564	**0.9328**	0.9375
DANRL-CA-CSM/Multilevel	**0.9575**	0.9506	0.9300	0.9374

⋆ We use red bold to highlight the best performance, and utilize black bold to show the performance comparison results between DANRL-CA-AM, and DANRL-CA-CSM, respectively. Significantly, there is the overlap between the red bold part and the black bold part.

Unlike the experimental results on the node classification task, the performance of M-NMF is always far better than that of LINE. However, DANRL-CA models still exhibit performance close to or better than ANRL-WAN most of the time. The above presents that M-NMF is not always suitable for any tasks, and proves the rationality of our models for community information modeling. Similarly, in most instances, the performance of the methods based on the structure and the attributes is far superior to those based on the structure, which demonstrates that the reasonable use of attribute information is also conducive to the link prediction task. Combined with all the above conclusions, the proposed DANRL-CA models achieve relatively good experimental results in most cases, demonstrating the effectiveness and scalability of our method.

Furthermore, as discussed in the node classification task, we can find similar conclusions from Table 10. The modularization of community detection methods makes the model flexible. The design of DANRL-CA-CSM is more conducive to produce distinguishable neighborhood information than DANRL-CA-AM if the network structure is more and more sparse, and the number of node attributes is small, which is clearly reflected in the PubMed dataset.

### 6.2 Brain Network

Previous studies Drakesmith et al. (2015) Akiki et al. (2018) have found that the missing or false edge could lead to an abnormal brain function which might cause a disease. Therefore, we here apply network representation learning to the field of brain network research. We implement the link prediction task on the brain network Fly-drosophila-medulla-1 without label information. Most algorithms which are compared in Section 6, are applied to the brain networks, except for the M-NMF and ANRL-WAN. Since the M-NMF needs the actual number of communities, which is not given in the brain network, and the ANRL-WAN will degenerate into DeepWalk when the attribute information is not considered. The results in Table 11 shows that our proposed models achieve better performance than the state-of-art methods in the brain network.

**TABLE 11:** Link prediction results on Fly-drosophila-medulla-1 dataset.

Datasets	Fly-Drosophila-Medulla-1
Evaluation	AUC
DeepWalk	0.6589
node2vec	0.6004
LINE	0.6073
SDNE	0.7961
M-NMF	—
ComVAE (Infomap)	0.6885
ComVAE (LPA)	0.6429
ANRL-WAN	—
DANRL-CA/Infomap	**0.8972**
DANRL-CA/LPA	0.7943
DANRL-CA/Multilevel	0.8024
DANRL-CA/NoCommunityInformation	0.8594

⋆ We use red bold to highlight the best performance.

Especially, de Haan *et al.* point out that brain networks have significant community structure characteristics de Haan et al. (2012). Hence, it is necessary to explore whether capturing community information is beneficial for learning node representations. We further apply our method to brain network without considering mining community information, and the result is given in the last row in Table 11. Compared with DANRL-CA/NoCommunityInformation, DANRL-CA/Infomap has better performance, while DANRL-CA/LPA and DANRL-CA/Multilevel obtain poor experimental results. The comparison results demonstrate that the validity of the mined community information will greatly promote the quality of node representations, and vice versa. To prove our idea, we then analyze the distribution of communities excavated by the three community detection algorithms, Infomap, LPA and Multilevel, respectively. We find that Infomap tends to mine small-scale community structures with nodes on the order of ten, LPA gathers about 92% of the nodes into a large community, and Multilevel prefers to explore community structures that are aggregated by nodes on the order of hundred. Significantly, Betzel et al. (2019) Betzel and Bassett (2017) show that small communities associated with functionally-specialized areas (the scale measurable with MRI) are ubiquitous in brain networks. The community division result of Infomap is closer to the actual structural definition of brain network than that of LPA and Multilevel, which is further verified in the performance comparison of ComVAE (Infomap) and ComVAE (LPA) in Table 11.

## 7 Conclusion

Researchers have found that many neuropsychiatric diseases (such as Alzheimer’s disease and schizophrenia) are associated with abnormal topological changes in brain structure and brain functional networks. Moreover, the development of the brain and the realization of cognitive tasks all depend on the interaction of neural activities between brain regions, which can be inferred by some edge prediction tasks, such as link prediction. The network representation learning can provide a new direction for brain network research and analysis. In the work, we propose a deep coupling DANRL-CA framework in the paper, which incorporates the community information and attribute semantics into NRL via deep neural networks. Specifically, DANRL-CA consists of neighborhood enhancement autoencoder module and community-aware skip-gram module, which are designed to preserve the second-order and higher-order proximity, respectively. For the processing of community information, we provide two solutions. DANRL-CA-AM model regards the community information as node attributes, while DANRL-CA-CSM model constructs a community similarity matrix on the observation that the community information can impose constraints from a high structure level on the node representations. We mine the community information and attribute semantics, which are integrated with the adjacency matrix as the input of our models. Then, we improve the accuracy of second-order proximity. Notably, we first verify the effect of our models on common datasets with attribute information and node label information. Next, we apply the proposed method to brain network and achieve excellent performance. We see that, on the one hand, a large number of experimental results prove the effectiveness and scalability of our method. Meanwhile, DANRL-CA-CSM can balance the effect of heterogeneous information, including the attribute semantics and community information, on network representation, and achieves better performance on the networks with sparse network structure and node attributes over DANRL-CA-AM. On the other hand, network representation learning plays an important role and significance in the study of brain networks, so there can be a lot of meaningful and valuable work to be done in the future, such as explorations that depends on specific tasks.

## Data Availability

The original contributions presented in the study are included in the article/supplementary material, further inquiries can be directed to the corresponding author.
